# Supplementary material for: Multi-omics analyses reveal that the gut microbiome and its metabolites promote milk fat synthesis in Zhongdian yak cows
Source: PeerJ. 2022 Dec 2;10:e14444. doi: 10.7717/peerj.14444 (PMC9744170; doi:10.7717/peerj.14444)
Supplement: Supplemental Information 13 [file peerj-10-14444-s013.zip › Web_Report/Metabolites_annotation/KEGG/meta_kegg_anno.pdf]

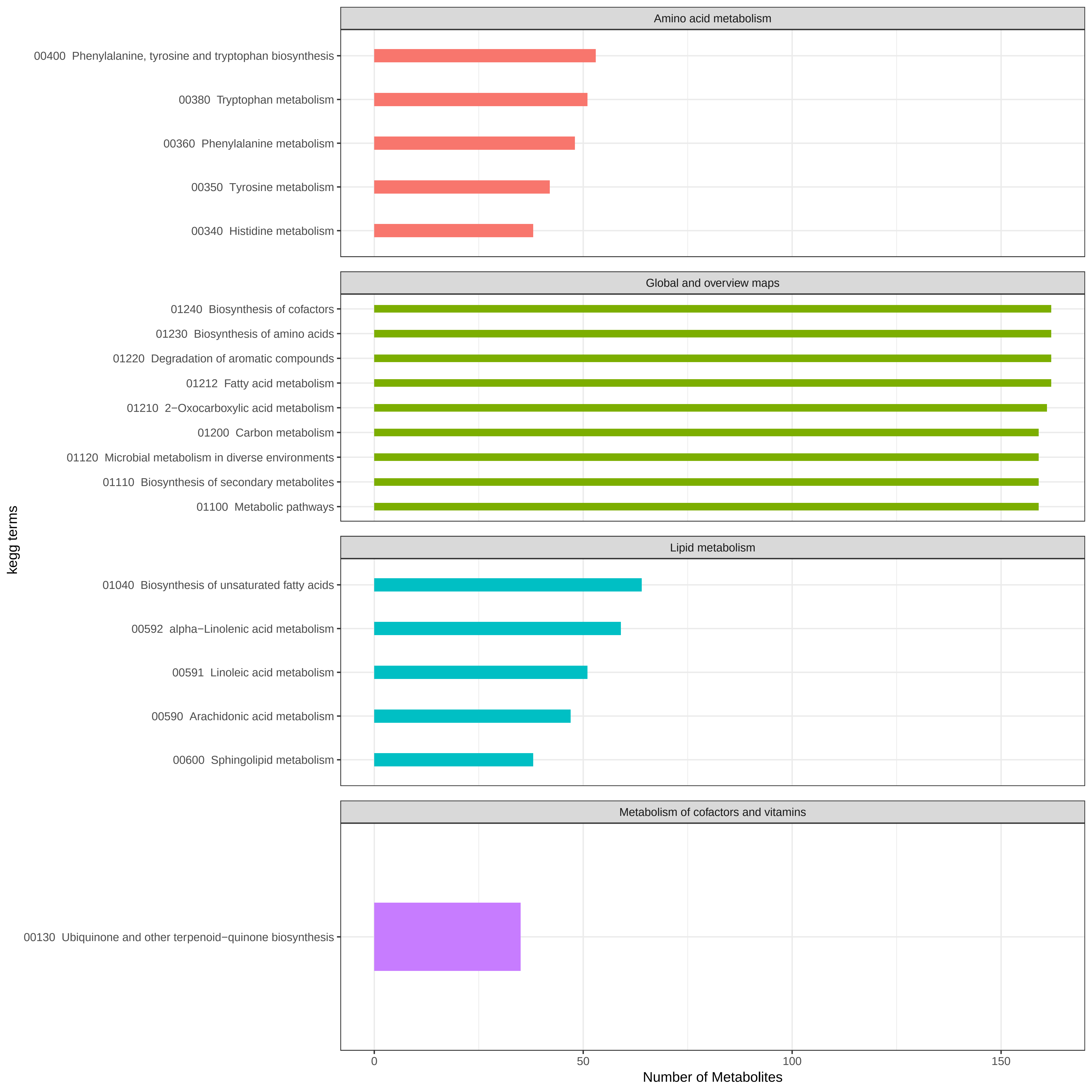

Amino acid metabolism
00400 Phenylalanine, tyrosine and tryptophan biosynthesis
00380 Tryptophan metabolism
00360 Phenylalanine metabolism
00350 Tyrosine metabolism
00340 Histidine metabolism
Global and overview maps
01240 Biosynthesis of cofactors
01230 Biosynthesis of amino acids
01220 Degradation of aromatic compounds
01212 Fatty acid metabolism
01210 2−Oxocarboxylic acid metabolism
01200 Carbon metabolism
01120 Microbial metabolism in diverse environments
01110 Biosynthesis of secondary metabolites
01100 Metabolic pathways
Lipid metabolism
01040 Biosynthesis of unsaturated fatty acids
00592 alpha−Linolenic acid metabolism
00591 Linoleic acid metabolism
00590 Arachidonic acid metabolism
00600 Sphingolipid metabolism
Metabolism of cofactors and vitamins
00130 Ubiquinone and other terpenoid−quinone biosynthesis
kegg terms
0
50
100
150
Number of Metabolites
